# Novel Respiratory Syncytial Virus A Genotype, Germany, 2011–2012

**DOI:** 10.3201/eid1906.121582

**Published:** 2013-06

**Authors:** Christiane Prifert, Andrea Streng, Christine D. Krempl, Johannes Liese, Benedikt Weissbrich

**Affiliations:** University of Würzburg, Würzburg, Germany

**Keywords:** respiratory syncytial virus, attachment protein G, epidemiology, RSV A genotype ON1, Germany, viruses, respiratory infections, RSV, genotype

**To the Editor:** Respiratory syncytial virus (RSV) is a major cause of severe respiratory disease in infants and elderly persons. RSV strains have been divided into 2 major antigenic groups (A and B), which are further divided into several genotypes. The main genetic and antigenic differences between genotypes are found within the 2 hypervariable regions of the attachment (G) glycoprotein. In 1999, a novel RSV B genotype, which contained a 60-nt duplication in the second hypervariable region of the G protein, was discovered in Buenos Aires, Argentina, and named BA (*1*). Since then, genotype BA has almost completely replaced other RSV B strains worldwide and has diversified into several subgenotypes (*2*). 

In February 2012, as part of routine RSV surveillance, we identified a novel RSV A genotype with a 72-nt duplication in the second hypervariable region of G, thus representing the first RSV A genotype with nucleotide duplications in the G gene. Shortly thereafter, circulation of this genotype was reported in Ontario, Canada, in 2010–11 and 2011–12, and the genotype was named ON1 (*3*). To investigate the frequency of genotype ON1 in Germany, we extended the molecular analysis of RSV strains from the previous 2 RSV seasons. The study was approved by the ethics committee of the medical faculty at the University of Würzburg, Germany.

From July 2010 through June 2011 and from July 2011 through June 2012, we identified 271 and 181 RSV-positive patients, respectively. Patients were identified from respiratory specimens sent by hospitals in Bavaria, Germany, for routine testing of respiratory viruses at the Institute of Virology and Immunobiology at the University of Würzburg. The mean age of all patients was 1.2 years (median 8.2 years; range 0.03–81.4 years), and 259 were male. Of the RSV-positive samples, 183 (67.5%) from season 2010–11 and 171 (94.5%) from season 2011–12 were analyzed by sequencing a fragment of ≈500 nt that encompassed the complete second hypervariable region of the G gene (*4*). Alignment with reference sequences and phylogenetic analyses were conducted by using MEGA 5.0 (*5*).

Molecular analysis of RSV-positive samples revealed that RSV A and B cocirculated during both seasons (98 A and 85 B during 2010–11; 99 A and 73 B during 2011–12). In accordance with previous reports (*2*), all RSV B strains from both seasons were identified as genotype BA. The novel RSV A genotype ON1 was not detected during 2010–11. However, 10 (10.1%) of 99 RSV A strains were assigned to genotype ON1 during 2011–12. All other RSV A strains of both seasons belonged to genotype GA2. An amino acid alignment of ON1 sequences is shown in the Figure. The duplication regions of 2 of the 10 ON1 strains contained 2-aa and 3-aa exchanges compared with the ON1 reference sequence (which has no exchanges) (*3*). Of note, an ON1 sequence from Japan with similar, partially even identical mutations was retrieved from GenBank (Figure). All mutations observed so far did not affect potential O-glycosylation sites.

**Figure:**
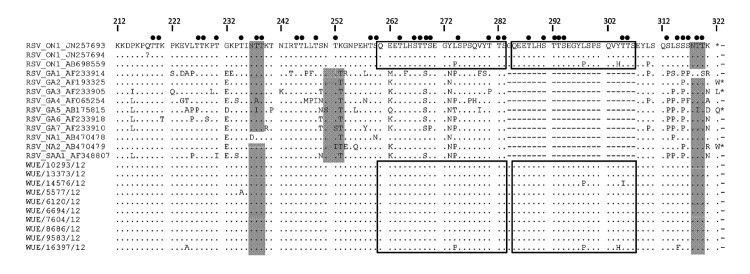
Amino acid sequence alignment of the second hypervariable region of the respiratory syncytial virus (RSV) G gene. RSV ON1 sequences and RSV A reference sequences in the upper part of the alignment are designated by GenBank numbers. ON1 sequences in the lower part were obtained in this study and are available at GenBank under accession nos. JX912355–JX912364. Black boxes indicate the duplicated region; black circles indicate potential O-glycosylation sites; and gray shading indicates potential N-glycosylation sites. Dots indicate nucleotide identities, dashes indicate adjustment of nucleotide insertions, and asterisks indicate stop codons. WUE, Würzburg.

Of the 99 patients with RSV A infection diagnosed during 2011–12, a total of 91 were hospitalized children. Genotype ON1 was identified in 7 (25.0%) of 28 children in intensive care units (ICUs) and in 2 (3.2%) of 63 children in other wards (p = 0.003, Fisher exact test). Children admitted to an ICU were younger (median 0.2 years) than those not in an ICU (median age 1.2 years; p<0.001, Mann-Whitney U test). An exploratory logistic regression analysis on ICU admittance, adjusted for age, confirmed a strong association between RSV genotype ON1 and ICU admittance (adjusted odds ratio 8.4; 95% CI 1.5%–46.6%; p = 0.015). However, this significant difference should be interpreted with caution for 2 reasons: 1) samples from patients in wards other than an ICU originated mainly in the Würzburg area, whereas samples from patients in ICUs were received from pediatric hospitals in various regions of Bavaria; 2) clinical information on patients not in ICUs was not available for assessment of whether the difference persisted when taking into account other risk factors for severe RSV disease.

In summary, the novel RSV A genotype ON1 containing a 72-nt duplication in the G gene was not found during 2010–11, but it constituted already 10.1% of all RSV A strains in a patient cohort from Bavaria, Germany, in the next season, 2011–12. In the context of the primary report of ON1 in Ontario, Canada (*3*), and the GenBank entry from Japan, our data suggest worldwide emergence of ON1. The almost complete worldwide replacement of circulating RSV B genotypes with the BA strain containing a comparable 60-nt duplication, which began in 1999, suggests that these duplications provide a selective advantage (*2*). Thus, molecular analysis of circulating RSV strains should be continued to determine whether ON1 has the potential to replace other RSV A strains in the years to come as did RSV B genotype BA during the past decade.
